# Faculty Onboarding for Workforce Readiness and Belonging

**DOI:** 10.7759/cureus.62856

**Published:** 2024-06-21

**Authors:** Melissa M Masaracchia, Scott D Markowitz, Norah R Janosy, Kim M Strupp

**Affiliations:** 1 Department of Anesthesiology, Pediatric Anesthesiology, Cohen Children’s Medical Center, Zucker School of Medicine at Hofstra/Northwell, Queens, USA; 2 Department of Anesthesiology, Washington University School of Medicine, St. Louis Children's Hospital, St. Louis, USA; 3 Department of Anesthesiology, Division of Pediatric Anesthesiology, Children's Hospital Colorado, University of Colorado, Denver, USA

**Keywords:** onboarding improvement, retention, new hire, orientation, faculty development programs, faculty development, onboarding assessment, onboarding buddy, enculturation, onboarding

## Abstract

Introduction: An academic anesthesiology department benefits from recruiting faculty from various centers, from new graduates to experienced clinicians. Two critical objectives for a department are getting the faculty members up-to-speed thoroughly and efficiently and retaining the faculty members to benefit from their contributions over time. Onboarding plays a pivotal role in meeting both objectives. A successful onboarding process is critical to the enculturation of new employees into an existing work environment. Organizations focusing on improving onboarding practices increase overall success, decrease attrition, and enhance member performance and satisfaction. In this study, we examine our onboarding practices and then create structured tools to improve our processes.

Methods: A survey gauging the effectiveness and satisfaction of our existing onboarding practices was administered to 11 faculty members hired between 2016 and 2018. Using feedback from the survey, our team identified critical components for improvement and quality measures for onboarding from before faculty arrival until after starting clinical duties. We also measured faculty satisfaction with the onboarding process at different time points. Updated onboarding practices targeting identified areas were implemented in one hiring cycle. Thirteen new faculty members hired over the course of the course of six months assessed the new system's effectiveness. The experience of the previous cohort was compared to the new cohort, highlighting the impact of their feedback on the onboarding process.

Results: Our new best practices model, implemented to address primary gaps in our system, has shown promising results. The post-intervention cohort reported more favorable responses to the process, suggesting a positive shift in the onboarding experience. Further free-text feedback included recommendations for additional updates, offering a proactive approach to continuous improvement.

Conclusion: A structured, feedback-responsive onboarding process improved the overall experience for new hires. While the response was overwhelmingly positive, further refinement with subsequent iterations is needed to continually improve this process.

## Introduction

Onboarding is a process that allows faculty to acquire the knowledge, skills, and cultural awareness to become influential members of an organization. This experience can have lasting impacts on employee satisfaction, improving fit within organizational culture, aligning resource use, and increasing overall employee success [1]. The onboarding process is also the first indicator of psychological safety in the workplace. This essential team element allows individuals to ask questions and take risks, leading to better outcomes and more fulfillment [2]. Not only is this process critical to the enculturation of new employees into an existing work environment, but onboarding is also necessary to provide general orientation, explain the execution of roles, and discuss organization relations.

In 2005, nearly 67% of companies failed to provide a formal onboarding process [3]. That number decreased rapidly to 24% in 2006 and is likely still low [4]. Companies focusing on improving their onboarding practices have demonstrated profound positive results, including improved employee performance and satisfaction, decreased attrition, and increased business success [5,6]. For example, Texas Instruments found that new hires reached "full productivity" two months earlier using formal onboarding practices. In doing the same, Bank of America reduced its executive failure rate from 40% to 12% [1]. Companies with immense hiring capabilities, like Microsoft, have teams dedicated to refining the onboarding process because these changes affect thousands of new hires each cycle [7]. However, these best practices have rarely been used within large healthcare organizations [8-10].

Healthcare systems are dynamic, and role adjustments are expected as clinical and administrative responsibilities vary over time. Onboarding programs should be capable of addressing the needs of new faculty in an ever-changing landscape. However, healthcare organizations need more guidance on how to develop and implement gold-standard onboarding practices. Because academia leverages significant resources, training, and relationships, onboarding represents a critical period for both the individual and the institution [11-13]. A successful experience can provide a stable base for new hires to find satisfaction and belonging, leading to increased psychological safety and productivity and higher levels of employee retention.

In a needs assessment survey of faculty at our institution, we found variability in applying baseline onboarding practices. Additionally, our institution has yet to assess the effectiveness of these processes. While efficient enculturation of new faculty helps maximize early and long-term success, creating a supportive and collaborative culture at the onset is a priority to optimize success for the individual and the team. Therefore, our objective was to examine our current onboarding process to identify gaps in the transition process and target these areas for subsequent improvement. Further, we aimed to create an onboarding best practices model that other healthcare organizations could broadly apply.

## Materials and methods

**Figure 1:**
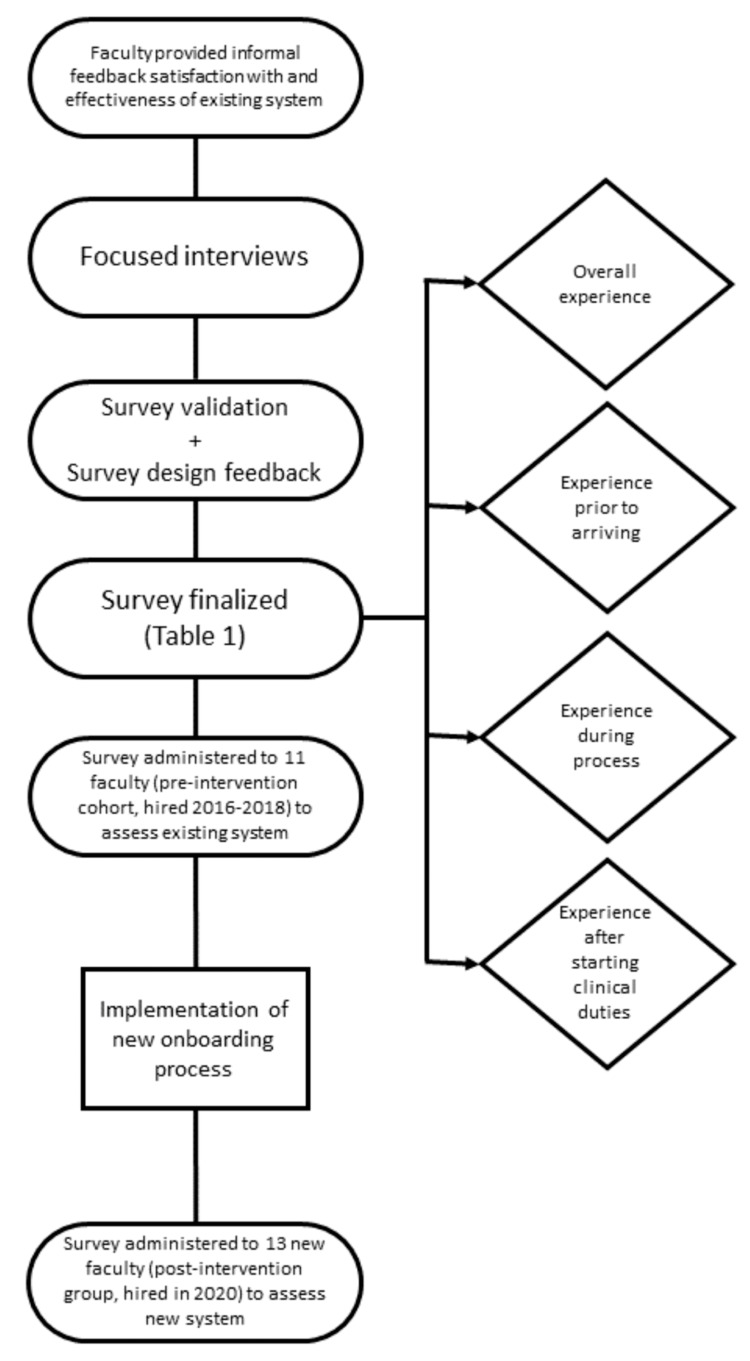
Flow diagram depicting the survey design, revision, and implementation. Four main domains were assessed prior to and after the implementation of new onboarding processes to determine their effectiveness: (1) overall experience, (2) experience before arriving, (3) experience during the process; and (4) experience after starting clinical duties

After obtaining approval from the Organizational Research Risk and Quality Improvement Review Panel at Children's Hospital Colorado, we assessed the existing onboarding process used by the division of pediatric anesthesiology. We developed preliminary survey questions using survey design principles outlined by Gelbach et al. [14]. Initially, one faculty member experienced with qualitative interviewing conducted interviews to assess face and content validity using cognitive interviewing techniques with two anesthesiologists and one anesthetist for language clarity, comprehension, and comprehensiveness. The survey included vital quality measures for onboarding events before arriving, during orientation, and after starting clinical duties. Feedback from faculty guided our changes to the initial survey, incorporating their concerns and supporting face and content validity (Figure 1).

The final survey (Table 1) targeted four main aspects of the onboarding process with more detail: (1) overall experience; (2) experience before arriving (important contacts, relocation, and credentialing/licensure information); (3) experience during onboarding (academic and clinical resources, schedules, and benefits); and (4) experience after starting clinical duties (electronic health record preparedness, access to medication-dispensing systems, phone-system orientation, familiarity with remote clinical sites). We administered the pre-intervention survey to 11 faculty members, including six physicians and five advanced practice providers (APPs), who were hired between 2016 and 2018. Survey questions utilized a Likert scale with values ranging from 1 to 5 (1=very dissatisfied, 5=very satisfied).

**Table 1:** Onboarding assessment survey used to evaluate and improve the onboarding process CHCO: Children's Hospital Colorado, APP: advanced practice providers

Please answer the following questions regarding your onboarding experience using the following scale	1=strongly disagree, 2=disagree, 3=neutral, 4=agree, 5=strongly agree	
Question		Area of assessment
1. Please select your faculty position:		Identification of target audience
Anesthesia APP		
Anesthesiologist		
Pain medicine physician		
Pain services APP		
Other (please specify)		
2. Overall, my onboarding and orientation experience was well-organized.		Communication
		Addressing expectations
		Providing necessary resources
		Timeliness of onboarding schedule
		Establishing a mentor connection
3. When considering your onboarding and orientation experience prior to arriving at CHCO, how satisfied were you with each of the following?		Communication
Contact information		
Relocation information		Addressing expectations
Credentialing and licensure information		
4. When considering your onboarding and orientation experience, after arriving at CHCO, how satisfied were you with each of the following?		Addressing expectations
Academic or office resources (computer, printer)		Providing necessary resources
Clinical and call schedule orientation		
Benefits orientation		
5. When considering your onboarding and orientation experience, after starting your clinical duties, how satisfied were you with each of the following?		Providing necessary resources
Electronic medical record preparedness		Established a mentor connection
Pivot (PCD/phone) orientation		
Comfort with various clinical sites		
Finding the bathroom? breakroom? stairs?		
Please provide a more elaborate response to the following free text questions:		
6. During the onboarding and orientation process, the following things were handled very well.		Communication
		Addressing expectations
		Providing necessary resources
		Timeliness of onboarding schedule
		Establishing a mentor connection
7. During the onboarding and orientation process, the following things could be handled better.		Communication
		Addressing expectations
		Providing necessary resources
		Timeliness of onboarding schedule
		Establishing a mentor connection
8. During the onboarding and orientation process, the following things were missing and should be considered for future onboarding/orientation.		Future iterative changes to process
9. Was shadowing during clinical care part of your orientation? (yes/no)		Communication
		Established a mentor connection
10. What else would you like us to know about your experience and the department's onboarding process?		Communication
		Addressing expectations
		Providing necessary resources
		Timeliness of onboarding schedule
		Establishing a mentor connection
11. If you are willing to provide additional feedback, please provide your contact information.		Future iterative changes to process

To use already established standards as a framework to revamp our existing onboarding model, we conducted a literature review to identify best practice guidelines for onboarding new faculty in an academic medical setting. We used keywords including onboarding, academic medicine, faculty development, orientation, and new hire to query PubMed and Google Scholar databases. These searches yielded detailed manuscripts about the process in other major industries, but only some applied to academic medical centers [1,7,11]. Finally, we interviewed administrative staff involved in the onboarding process to gauge areas needing improvement.

Based on the results of the initial survey, two actionable items were targeted and served as the foundation for the new onboarding program: (1) a reproducible, standardized checklist of required elements with correlating resources necessary for orientation completion and (2) an assigned onboarding faculty liaison as part of the "buddy system" who is well-versed in department policies and standards. New faculty hires were allowed 30 days to complete tasks using this system. Assigned and trained "onboarding buddies" were asked to check in weekly to ensure accountability.

Our new best practices model was implemented over the following hiring cycle with the intention of measuring the effectiveness of the changes included in the new onboarding process. A total of 10 physicians and three APPs were hired between May 1, 2020, and October 1, 2020. We sent our onboarding survey to these 13 new hires within 60 days of their start date, and 12 responded with a response rate of 92%. The surveys were anonymous. A two-sample t-test was performed to compare mean satisfaction levels between the pre-intervention and post-intervention groups.

## Results

The pre-intervention group consisted of all 11 new faculty members hired between 2016 and 2018: six physicians and five APPs. The post-intervention group included all 13 new hires from 2020: 10 physicians and three APPs. All members completed the survey successfully, except for one APP in the post-intervention group, for an overall response rate of 96% (100% pre-intervention and 92% post-intervention; Table 2).

**Table 2:** Pre-intervention and post-intervention respondents and response rate to our satisfaction surveys APPs: advanced practice providers

Faculty position	Pre-intervention surveyed	Pre-intervention responses	Pre-intervention response rate	Post-intervention surveyed	Post-intervention responses	Post-intervention response rate
Physicians	6	6	100%	10	10	100%
APPs	5	5	100%	3	2	67%
Total	11	11	100%	13	12	92%

Table 3 presents the pre-intervention survey results. A score of 1 indicates being "very dissatisfied," while a score of 5 indicates being "very satisfied" with each element of the onboarding experience.

**Table 3:** Results of the pre-intervention survey demonstrating the number and percent of respondents for each satisfaction score

Pre-intervention	Number (percent) of respondents per satisfaction score					
Category	1 – very dissatisfied	2	3	4	5 – very satisfied	No response
Organization	0 (0%)	2 (17%)	1 (9%)	6 (55%)	2 (18%)	0 (0%)
Contact information	0 (0%)	0 (0%)	2 (17%)	3 (27%)	6 (55%)	0 (0%)
Relocation information	1 (9%)	2 (17%)	2 (17%)	2 (17%)	4 (36%)	0 (0%)
Credentialing and licensure information	1 (9%)	0 (0%)	2 (17%)	3 (27%)	5 (45%)	0 (0%)
Academic or office resources	1 (9%)	0 (0%)	1 (9%)	3 (27%)	5 (45%)	1 (9%)
Clinical and call schedule orientation	0 (0%)	0 (0%)	3 (27%)	3 (27%)	4 (36%)	1 (9%)
Benefits orientation	0 (0%)	0 (0%)	0 (0%)	4 (36%)	6 (55%)	1 (9%)
Electronic medical record preparedness	0 (0%)	0 (0%)	1 (9%)	4 (36%)	6 (55%)	0 (0%)
Phone orientation	2 (17%)	1 (9%)	1 (9%)	1 (9%)	4 (36%)	2 (18%)
Comfort with various clinical sites	1 (9%)	1 (9%)	1 (9%)	5 (45%)	3 (27%)	0 (0%)
Finding the bathroom? Breakroom? Stairs?	0 (0%)	0 (0%)	1 (9%)	3 (27%)	6 (55%)	1 (9%)

Based on survey results and a thorough review of the current onboarding model, we found that essential elements critical to an effective transition process were missing from our system. Gaps included inconsistencies in the orientation process, highlighting a need for a reproducible onboarding experience. Aligned with this deficiency in uniformity was the absence of standardized methods to assess orientation completion. Additionally, when new hires used faculty orientation handbooks for guidance, they were found to contain outdated or inaccurate information. Satellite location-specific orientation needs were also not addressed adequately. Finally, key individuals well-versed in department policies and onboarding processes could have helped the overall experience. Survey respondents identified two individuals (one anesthesiologist and one anesthetist) critical to the onboarding process. When one was absent from the process, the respondents' overall experience suffered.

As mentioned previously, two areas for improvement served as the foundation for the new onboarding program: providing a standardized checklist with required tasks, correlating resources, and assigning individual faculty liaisons (buddies) knowledgeable about department policies and standards (Table 4).

**Table 4:** An example of an onboarding checklist we used to improve our onboarding processes LOO: letter of offer, HR: human resources, CHCO: Children's Hospital Colorado, BLS: basic life support, ACLS: advanced cardiac life support, PALS: pediatric advanced life support, DEA: Drug Enforcement Administration, CARS: common access request system, CU: University of Colorado, APP: advanced practice provider, MRI: magnetic resonance imaging, CAA: certified anesthesiologist assistant, CRNA: certified registered nurse anesthetist, IT: information technology

Anesthesia onboarding checklist	
Credentialing	Ensure the new faculty member has formally applied online through the university
	An initial LOO will be emailed to the new hire. Once the offer is accepted, all parties will sign the formal LOO and mail the offer packet to the new hire from university HR
	Once the faculty member has accepted the position, begin the process for children’s hospital credentialing
	The CHCO credentialing packet is sent electronically from the medical staff member to the faculty member via email. The packet clearly states that it may take up to 120 days for privileges to be approved
	On the 2^nd^ Tuesday of every month, a new hire’s file must be completed and submitted to the credentialing office
	On the 3^rd^ Tuesday of every month, the credentialing committee meets to approve privileges/credentialing for new hires to begin on the 1^st^ day of the coming month (privileges cannot be granted any day other than the 1^st^)
	All new faculty members are required to have both north campus and south campus privileges
	The following documents are required to create the billing account and profile: Color copy of driver’s license, state medical license, master of science or doctor of medicine diploma, board certification, fellowship and residency completion certificates, BLS/ACLS/PALS, DEA, and children’s application and consent must be hand signed and dated per the university. Signatures must be within 45 days of the start date
	Request a copy of malpractice insurance
Departmental tasks	Assign a “buddy” before their start date
	Personal mailbox
	Phone assigned
	Give phone directory cards to attach to the badge
	Professional expense account
	QGenda (scheduling) accounts
	Create account
	Orient with faculty contact
	Assign office to new attending physicians
	Add email addresses to the department and division distribution lists
	Omnicell access
	Once the faculty member is privileged, submit a CARS request for access. Once access is granted, they will log in with their children’s ID and create a password. The faculty member will need to meet with an attending or anesthetist to guide them
Orientations: all scheduled orientations should be completed within 2 weeks of the NFM start date	University orientation is from 8:30-4:00. Dates and locations vary
	CU med orientation. Dates and times vary
	CHCO orientation 8:30-11:30 am (takes place on the 1^st^ and 15^th^ of every month)
	Set up through the med staff department. Professional headshot, badge, parking, lab coat fitting, policies and procedures, target zero, hospital tour
	CHCO APP same day as CHCO orientation 1:00-3:30 pm
	Set up through medical staff admin
	EPIC training 8:00-4:30 pm
	MRI safety training
	ISTAT training
	Ensure the new faculty member has computer/outlook access following CHCO orientation
	Call responsibilities and night/weekend coverage
30-minute meetings	Anesthetists and Attendings meet with the Lead anesthesia tech for orientation to the anesthesia tech team
	Attendings and anesthetists meet with the division chief
	Attendings meet with the director of operations to discuss clinical operations
	Attendings meet with the clinical director to discuss policies and procedures
	If the faculty member has IT or EPIC questions, meet with the director of IT
	Anesthetists should meet with the anesthetist liaison within their first few weeks
	Attendings meet with the north campus medical director
	Attendings meet with the south campus medical director
	Attendings and anesthetists meet with the human resources administrator to review policies
	Meet with the chair of finance and administration
	Attendings meet with the department chair to touch base and for anesthetists to meet and greet
Shadowing	Anesthetists
	The CAA/CRNA will shadow experienced anesthetists and attendings in all locations at the Anschutz campus for the first two weeks. The admin schedulers will assign them. The anesthetist chief will check in with them to see when they are comfortable enough to be on their own
	Attendings
	The attending will be scheduled to shadow a senior doctor in all locations at Anschutz for the first one to two weeks. The admin schedulers will assign them. The director of operations and division chief will touch base with the attending during the first few clinical weeks

Post-intervention survey results are presented in Table 5.

**Table 5:** Results of the post-intervention survey demonstrating the number and percent of respondents for each satisfaction score

Post-intervention	Number (percent) of respondents per satisfaction score				
Category	1 – very dissatisfied	2	3	4	5 – very satisfied
Organization	1 (8%)	0 (0%)	1 (8%)	4 (33%)	6 (50%)
Contact information	0 (0%)	0 (0%)	0 (0%)	3 (25%)	9 (75%)
Relocation information	0 (0%)	0 (0%)	1 (8%)	5 (42%)	6 (50%)
Credentialing and licensure information	0 (0%)	1 (8%)	0 (0%)	3 (25%)	8 (67%)
Academic or office resources	0 (0%)	1 (8%)	1 (8%)	5 (42%)	5 (42%)
Clinical and call schedule orientation	0 (0%)	1 (8%)	0 (0%)	3 (25%)	8 (67%)
Benefits orientation	0 (0%)	0 (0%)	1 (8%)	6 (50%)	5 (42%)
Electronic medical record preparedness	0 (0%)	1 (8%)	0 (0%)	2 (17%)	9 (75%)
Phone orientation	0 (0%)	0 (0%)	2 (17%)	3 (25%)	7 (58%)
Comfort with various clinical sites	1 (8%)	0 (0%)	1 (8%)	1 (8%)	9 (75%)
Finding the bathroom? Breakroom? Stairs?	0 (0%)	1 (8%)	0 (0%)	1 (8%)	10 (83%)

**Figure 2:**
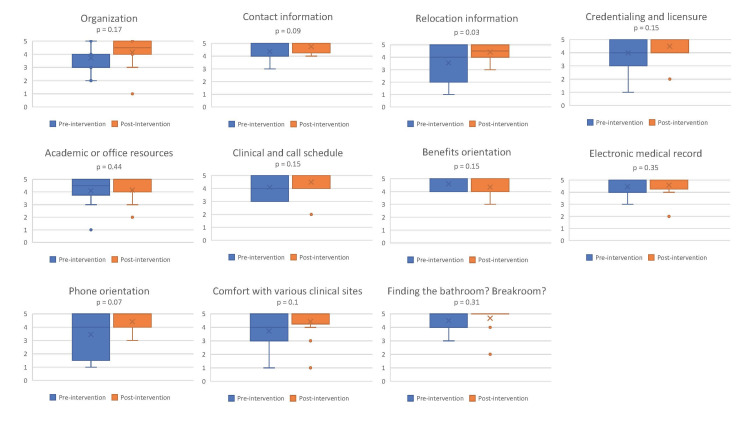
Box and whisker plots demonstrating the spread and dispersion of satisfaction scores. P-values were calculated using two-sample t-tests

While a two-sample t-test only demonstrated statistical significance between the pre-intervention and post-intervention groups (p<0.05) for the relocation information category, mean satisfaction ratings increased in 10 out of 11 categories (Figure 2). Looking at the box and whisker plots, the data was more narrowly dispersed at a higher interval for all the questions except one, benefit orientation. Benefits orientation was also the one category that scored lower in the post-intervention group; this orientation is managed outside the department by central human resources from the affiliated university.

Most respondents in the post-intervention cohort were satisfied with the new onboarding experience (83%). This satisfaction was especially favorable for the orientation processes occurring after arrival. For example, 91.7% of respondents were either satisfied or very satisfied with clinical and call schedule orientation, the explanation of benefits, and feeling prepared to use the electronic medical record. Furthermore, most new hires felt that the onboarding process addressed many of the items on the system checklist (badging, parking access, and Omnicell access) and felt comfortable finding key locations after only one week. Seven faculty members had trained at this institution and were still offered shadowing experiences but declined due to self-expressed familiarity with all anesthetizing locations.

The post-intervention group also completed a free-text comment section addressing the onboarding processes that went well. Logistically, respondents indicated they found the early orientation timing valuable and had a solid explanation of the benefits. Themes in responses aligned with psychological safety and included reports of feeling comfortable asking questions and feeling notably well-supported by their paired "buddy" and staff in general. Additionally, several comments noted that having access to meetings and discussions with leadership added to the positives of the onboarding experience.

For the free-text comment section regarding areas for improvement, common themes focused on system issues that we should address during the next iteration of onboarding practices. Responses were typically logistical, including challenges with hospital-issued phones and accessing the electronic health record as faculty for former trainees. Some needed scheduling and assignment clarification, which is an opportunity for better communication. Overall, the responses were positive, with one respondent stating, "Given that I have had a few onboarding experiences, I think this was one of the better experiences."

## Discussion

Clinical faculty recruitment and retention is essential in all departments, and current staffing challenges make this critical in anesthesiology [15]. Effective onboarding is crucial to employee satisfaction and increasing early productivity [16]. A large body of literature highlights the successes of onboarding practices in the private industry, while there is limited research on how to achieve the same success in healthcare systems [7]. Only a few healthcare departments have described their onboarding experiences [10,17-19]. They identified critical components for faculty onboarding and retention. Hebert described an onboarding process for new hire pathology residents where they were given access to baseline didactic curriculum before starting, were enculturated into the work ethos at the hospital, and were given a social welcome to the area [10]. Smith-Miller et al. used survey methods to examine experienced nurses' onboarding experiences and frame the findings within the organizational socialization domain [17]. Bethel et al. assessed the satisfaction of travel nurses with onboarding practices [18]. Scott et al. recommended onboarding practices during the COVID-19 pandemic, including strengthening new hires' knowledge, confidence, well-being, and social connections [19]. Constantly adapting onboarding experiences to new situations is essential to a successful process. To our knowledge, no other onboarding programs have undergone the evaluation and subsequent improvement process utilized by our team.

To improve the enculturation and retention of our new faculty and identify gaps to accelerate their successful launch in the department, we sought to measure current processes and identify recommendations for improvement. If our interventions positively impact these short-term measures, they can be examined with longer-term outcomes as a potential surrogate marker for positive culture and faculty retention.

Beginning with a needs assessment, we identified areas for improvement, including the need for an onboarding checklist and the identification of personnel who can orient new hires to successful local practices. Using a literature review of best practices, we developed an initial survey and structured interview questions to obtain faculty feedback on processes. Using feedback and best practices, we created and instituted a standardized onboarding process for new hires that addressed identified gaps. This updated onboarding approach improved overall satisfaction.

While much progress was made with the new process, our standard post-onboarding assessment identified some areas for improvement. When our global issues had been successfully addressed, other challenges appeared more apparent. This suggests that complex systems can be overwhelmed by multiple problems, and an inclination may be to focus on the major sources of dissatisfaction. Therefore, improving our process still offers an ongoing opportunity for further refinement.

We recognize several limitations of this study. Our sample size is small. As a department with under 100 faculty members, there is considerable variation in the number of faculty hired annually. We examined the process for one cycle and then adjusted processes using the updated feedback received. The onboarding practices are ever-evolving, so each change uses only one hiring cycle. Since our sample size was small, it likely accounts for the inability to show statistical significance in satisfaction scores between pre- and post-intervention. Our small sample size is affected by our low faculty turnover. In fact, our faculty turnover rate was 5-6% between 2019 and 2020 (5 out of 94 and 6 out of 95, respectively). Additionally, our study is limited by the pre- and post-intervention groups representing different cohorts. Our proportion of anesthetists was 45% of the total study subjects in the pre-intervention group, while anesthetists only represented 23% of the post-intervention group.

While the new onboarding practices were designed to guide new hires through the initial orientation process for up to 60 days, we did not assess their longer-term impact. The focus of an ongoing study will be connecting faculty satisfaction with onboarding to measures of early clinical effectiveness, enculturation, and retention.

With this in mind, future efforts should aim to determine how onboarding processes can be modified to improve the long-term success of faculty members in clinical effectiveness and efficiency, academic productivity, personal well-being, and organizational impacts, including retention of faculty members, recruitment, and reputation for a positive culture.

## Conclusions

Current challenges in academic medicine and anesthesiology to recruit and retain faculty are on the minds of department chairs as well as hospital and health system leadership. Organizational research demonstrates the positive impact of effective onboarding processes on new hires’ performance and retention. While some studies have examined this in the academic medical center, we have sought to introduce a process to continually improve onboarding processes with the intent to affect faculty satisfaction and to impact culture, retention, early efficiency in clinical areas, and longer-term effectiveness in all academic missions. In summary, medical systems are ever-changing landscapes requiring constant assessment. For a department to succeed, the practices needed to assimilate new hires should be adaptable. New hire onboarding is critical for enculturation into an organization, and consistent processes to onboard faculty are important. Our implementation of a structured, feedback-responsive onboarding process improved the overall experience for our new hires. Satisfaction scores were high, and further opportunities for refinement were identified through this process. Work to continually optimize this process is ongoing. We hope that our revamped onboarding process will help create an environment for employee success.

## Appendices

**Table 6:** Onboarding assessment survey administered to faculty to assess the onboarding process PCD: personal communication device

Please answer the following questions regarding your onboarding experience using the following scale	1=strongly disagree, 2=disagree, 3=neutral, 4=agree, 5=strongly agree
Question	
1. Please select your faculty position	
Anesthesia advanced practice provider	
Anesthesiologist	
Pain medicine physician	
Pain services advanced practice provider	
Other (please specify)	
2. Overall, my onboarding and orientation experience was well-organized	
3. When considering your onboarding and orientation experience prior to arriving at CHCO, how satisfied were you with each of the following?	
Contact information	
Relocation information	
Credentialing and licensure information	
4. When considering your onboarding and orientation experience, after arriving at CHCO, how satisfied were you with each of the following?	
Academic or office resources (computer, printer)	
Clinical and call schedule orientation	
Benefits orientation	
5. When considering your onboarding and orientation experience, after starting your clinical duties, how satisfied were you with each of the following?	
Electronic medical record preparedness	
Pivot (PCD/phone) orientation	
Comfort with various clinical sites	
Finding the bathroom? breakroom? stairs?	
Please provide a more elaborate response to the following free text questions:	
6. During the onboarding and orientation process, the following things were handled very well	
7. During the onboarding and orientation process, the following things could be handled better	
8. During the onboarding and orientation process, the following things were missing and should be considered for future on-boarding/orientation	
9. Was shadowing during clinical care part of your orientation? (yes/no)	
10. What else would you like us to know about your experience and the department's onboarding process?	
11. If you are willing to provide additional feedback, please provide your contact information	
